# Echocardiographic measurements of epicardial adipose tissue and comparative ability to predict adverse cardiovascular outcomes in patients with coronary artery disease

**DOI:** 10.1007/s10554-018-1360-y

**Published:** 2018-05-02

**Authors:** Julieta D. Morales-Portano, Juan Ángel Peraza-Zaldivar, Juan A. Suárez-Cuenca, Rocío Aceves-Millán, Lilia Amezcua-Gómez, Carlos H. Ixcamparij-Rosales, Rafael Trujillo-Cortés, Rogelio Robledo-Nolasco, Paul Mondragón-Terán, Rebeca Pérez-Cabeza de Vaca, Rolando Hernández-Muñoz, Alberto Melchor-López, Mani A. Vannan, Alberto Francisco Rubio-Guerra

**Affiliations:** 1 Departments of Echocardiography, Hemodynamics and Cardiology. Centro Médico Nacional “20 de Noviembre”, ISSSTE, Mexico City, Mexico; 2 Department of Clinical Research, Centro Médico Nacional “20 de Noviembre”, ISSSTE, 502, San Lorenzo, Col. Del Valle; Del. Benito Juárez, 03100 Mexico City, Mexico; 3 Department of Internal Medicine, H.G.Z. No. 58 and No. 8, Social Security Mexican Institute, Mexico City, Mexico; 4 Mexican Group for Basic and Clinical Research in Internal Medicine, Mexico City, Mexico; 5 Coordination of Research, Centro Médico Nacional “20 de Noviembre”, ISSSTE, Mexico City, Mexico; 6 Departamento de Biología Celular y Desarrollo, Instituto de Fisiología Celular, Universidad Nacional Autónoma de México (UNAM), 04510 Mexico City, CDMX Mexico; 7 Department of Internal Medicine, H.G. Xoco, H.G. Ticomán SSDF, Mexico City, Mexico; 8 Cardiovascular Imaging, Piedmont Heart Institute, Piedmont Atlanta Hospital, Atlanta, GA USA

**Keywords:** Echocardiography, Epicardial adipose tissue, Syntax score, Left ventricle ejection fraction, Major adverse cardiovascular events, Coronary artery disease

## Abstract

The present study aimed to compare echocardiography measurements of epicardial adipose tissue (EAT) thickness and other risk factors regarding their ability to predict adverse cardiovascular outcomes in patients with coronary artery disease (CAD). Outcomes of 107 patients (86 males, 21 females, mean age 63.6 years old) submitted to diagnostic echocardiography and coronary angiography were prospectively analyzed. EAT (measures over the right ventricle, interventricular groove and complete bulk of EAT) and left ventricle ejection fraction (LVEF) were performed by echocardiography. Coronary complexity was evaluated by Syntax score. Primary endpoints were major adverse cardiovascular events (MACE’s), composite of cardiovascular death, myocardial infarction, unstable angina, intra-stent re-stenosis and episodes of decompensate heart failure requiring hospital attention during a mean follow up of 15.94 ± 3.6 months. Mean EAT thickness was 4.6 ± 1.9 mm; and correlated with Syntax score and body mass index; negatively correlated with LVEF. Twenty-three cases of MACE's were recorded during follow up, who showed higher EAT. Diagnostic ability of EAT to discriminate MACE's was comparable to LVEF (AUROC > 0.5); but higher than Syntax score. Quartile comparison of EAT revealed that measurement of the complete bulk of EAT provided a better discrimination range for MACE's, and higher, more significant adjusted risk (cutoff 4.6 mm, RR = 3.91; 95% CI 1.01–15.08; p = 0.04) than the other risk factors. We concluded that echocardiographic measurement of EAT showed higher predicting ability for MACE’s than the other markers tested, in patients with CAD. Whether location for echocardiographic measurement of EAT impacts the diagnostic performance of this method deserves further study.

## Introduction

Epicardial adipose tissue (EAT), a store of visceral fat situated between the myocardium and pericardium, correlates with the extension and severity of coronary artery disease (CAD) and could be relevant for its local induction and/or progression [1, 2]. The pro-inflammatory mRNA profile in EAT is comparable to that in the omental adipose tissue [3], while EAT thickness is associated with pro-atherogenic biomarkers, nitric oxide and malondialdehyde (MDA), as previously reported by our group [4]. Therefore, EAT plays a role as a local inflammatory store in patients with CAD.

There is growing interest in the imaging assessment of EAT. Multislice computed tomography is an accurate method to measure the EAT bulk with high spatial resolution, and the possibility to evaluate in turn coronary atherogenesis and calcification [5, 6]. Echocardiography represents a useful method to measure of EAT, although clinical relevance of measurements at different anatomical locations is not clear. Echocardiography determination of EAT thickness provides advantages like: (1) safe, easily reproducible, and non-invasive method which can be routinely performed [6]; (2) echocardiography measure of EAT independently correlates with CAD [2, 7] and with plaque vulnerability [8, 9], in the context of acute coronary syndrome and chronic unstable angina. Based on such properties, potential ability of echocardiography measured EAT to predict future atherogenic-related adverse events has been explored [9–11].

Several studies have shown that cardiometabolic risk reflected by elevated EAT parallels the increase in other factors like the left ventricular mass and other components of the metabolic syndrome [12, 13]. Regarding prediction of major adverse cardiovascular events (MACE's), risk markers like EAT, Syntax score, LVEF and BMI have been characterized, and comparable results have been found by separate studies [9, 10, 14–18].

However, no study has specifically being designed to compare their predictive ability for MACE's, including echocardiography-determined EAT at different anatomical locations. Therefore, the present study was aimed to compare echocardiography measurements of EAT thickness, Syntax score, LVEF and BMI regarding their ability to predict MACE’s in patients with CAD.

## Methods

### Study population

This was an observational, longitudinal, single-center study carried out at the National Medical Center ‘20 de Noviembre’ ISSSTE, a tertiary referral hospital in Mexico City; following referral by health-care units that serve the general population. We initially included 118 consecutive patients > 18 years old with angina, in the absence of pericardial effusion, previous ICP, coronary artery bypass grafting or aortic and mitral valve repair or replacement, due to the possibility that EAT had been manipulated during the surgical procedure; who were submitted to diagnostic echocardiography and coronary angiography between September 2013 and February 2016, due to suspected CAD, according to the current guidelines [19]. Eleven patients were excluded due to inadequate acoustic screen during echocardiography, incomplete data and/or were lost at follow-up. Final study population was constituted by 107 patients. The study complied with the ethical guidelines of the 1964 Declaration of Helsinki and its later amendments, and was approved by the local Institutional Committees of Research, Ethics and Biosafety (Protocol ID No. 403.2013). All participants provided written informed consent.

### Study protocol

Coronary angiography, CAD characterization and classification of angina were performed at study enrollment. Echocardiography was performed within the first 48 h of hospital admission. At the same time the following demographic and anthropometric data were acquired: age, sex, current smoking status, BMI [calculated as weight/height^2^ (kg/m^2^)] and obesity (defined as a BMI ≥ 30 kg/m^2^). High blood pressure was defined as blood pressure ≥ 140/90 mmHg, when blood pressure was the mean of three readings taken with intervals of 5 min within each reading, and obtained while the patient was in a seated position, or treatment with antihypertensive agents. Dyslipidemia was defined as one or more of the following conditions: (1) total cholesterol ≥ 200 mg/dL; (2) LDL cholesterol ≥ 100 mg/dL; (3) HDL cholesterol < 40 mg/dL; or (4) triglycerides ≥ 150 mg/dL; or hyperlipidemia treatment. Diabetes mellitus was defined according to the guidelines from the American Diabetes Association with one of the following conditions (repeated for confirmation at a separate date): (1) Hb A1C ≥ 6.5%; (2) fasting glucose ≥ 126 mg/dL; or (3) 2-h plasma glucose ≥ 200 mg/dL during an oral glucose tolerance test. History of CAD was obtained from the clinical record of each patient. All patients received standard recommendations including medications and life-style changes to limit risk factors for CAD, according to the AHA/ACC guidelines. Patients were periodically followed up at a clinic visit every 3 months or were telephonically contacted in case of being residents of any state out of Mexico City.

### Echocardiographic parameters, LVEF and EAT

Echocardiography was performed using a Philips iE33 cardiac ultrasound system (Philips Medical Systems, Andover, MA, USA) and a Phillips iE33 S5-1 Transducer (Royal Philips, Amsterdam, The Netherlands). Echocardiographic parameters like E/A, E/Ea and Ventricle Mass (g/m^2^) were measured according to the guidelines of the American Society of Echocardiography. Left ventricle ejection fraction (LVEF) was measured using a modified Simpson method.

To determine EAT thickness, two-dimensional, M-mode and Doppler transthoracic echocardiography were performed in the left lateral position. EAT thickness was defined as the space between the visceral layer of the pericardium and the outer border of the myocardium, which registered on the standard parasternal longitudinal axis and transverse images. The aortic ring was used as the anatomical reference for the large axis parasternal view, and the papillary muscles were used for the short axis. The images were acquired at the end of systole, over ten cardiac cycles. EAT thickness was evaluated at different locations: EAT was measured on the free wall of the right ventricle from both parasternal long axis view (along the midline and perpendicular to the aortic annulus) and short-axis views at mid ventricle (perpendicular to the interventricular septum at midchordal and tip of the papillary muscle). In all of the cases, reproducibility of the measurement was validated using acceptable intraclass correlation coefficients (agreement) for inter-observer reliability.

### Coronary angiography, Syntax score and angina classification

Coronary angiography and stenting were performed as they were clinically indicated. The procedure was carried out according to standard techniques using radial approach. Evaluable vessels were larger than 1.5 mm and significant CAD was considered if luminal stenosis was > 50%. Coronary stenting was performed according to standard techniques, using drug-eluting stents in all cases (the drug eluted was everolimus in 50% of the cases, and zotarolimus in the remaining cases). The mean number of stents applied was one per vessel, while the size and length of the stent was determined by the cardiology expert, being 3 mm diameter, and 20 mm length stents the most frequently used. Standard dual antiplatelet therapy previous to PCI consisted of aspirin 150 mg/day and clopidogrel 75 mg/day in patients with chronic stable angina. Patients with acute coronary syndrome received an aspirin loading dose of 300 mg and a clopidogrel loading dose of 600 mg immediately before PCI. After PCI, all patients continued dual antiplatelet therapy, with clopidogrel discontinuation after 1 year. Syntax score was calculated according to classical description [20], using dedicated software (http://www.syntaxscore.com/calculator/start.htm). In all cases, coronary artery luminal narrowing was visually evaluated by two independent researchers, as recommended by the American Heart Association/American College of Cardiology. Angina intensity was evaluated according to the Canadian Cardiovascular Society (CCS) score [21].

### Follow up and study endpoints

Follow-up was conducted every three months after 2 years, either via telephone interview or through written reports from programmed medical evaluations (all subjects agreed to be contacted and to provide information during written consent). If telephone contact was not possible or physician visit were delayed > 2 months, the endpoints were verified through an authorized person previously designed. Primary endpoints consisted of MACE’s, including: (1) cardiovascular death, (2) myocardial infarction, defined as the presence of clinical signs with concomitant increases in CK-MB and troponin higher than 3 times the upper reference values with a previous determination within the reference values; (3) unstable angina, prompting an unscheduled visit to an emergency department within 24 h, (4) intra-stent re-stenosis as demonstrated by coronary angiography, (5) episodes of decompensate heart failure requiring hospital attention.

### Statistics

The study was designed to evaluate the ability of EAT thickness to predict MACE’s in patients with CAD, as compared with other markers. For descriptive statistics, continuous variables are presented as mean ± SD, and categorical variables are presented as *n* (%) prevalence. Data distributions for the variables were estimated using Kolmogorov–Smirnov test. Pearson’s correlation coefficient was applied to determine the relationship between EAT and variables like age, BMI, LVEF y and Syntax score. To evaluate the ability of EAT to predict MACEs, the mean EAT thickness from cases developing MACEs was compared with mean EAT from those without MACEs, using unpaired Student’s *t* tests. Likewise, diagnostic ability to predict MACEs was estimated using a receiver operating characteristic curve and the area under the ROC curve (AUC). Difference in the range distribution of MACE’s according to the location of EAT measure is shown through the comparative number of identifiable MACE’s at each EAT quartile, either measured on the ventricle free wall or measured as the complete bulk of EAT. Regression analyses (modeling through the ‘enter’ method) were performed to determinate the probability of MACE’s linked to EAT thickness or the other markers, as well as the weight of variables of potential confusion. The relative risk and 95% confidence intervals (CIs) are shown. All statistical analyses were performed using SPSS software, version 23.0 (SPSS Inc., Chicago, IL, USA) for Windows®, and p values ≤ 0.05 (2-tailed) were considered to be statistically significant.

## Results

The study population was constituted by 107 patients, mean age was 63.6 years old (86 males, 21 females) whose demographic and clinical characteristics are summarized in Table 1. High blood pressure and dyslipidemia were significantly prevalent. Half of patients reported previous CAD event and current medical consultation was because of chronic stable angina; most of them at class II from the Canadian Cardiovascular Society functional classification.

**Table 1:** Demographic and clinical characteristics (n = 107)

Age (years)	63.6 ± 9.67
Male [n (%)]	86 (80.4)
BMI	27.7 ± 3.77
Smoke	52 (48.6)
Co-morbidity	
High blood pressure	77 (72.0)
Dyslipidemia	65 (60.7)
Type 2 diabetes mellitus	54 (50.5)
Obesity	25 (23.4)
History of CAD	52 (48.6)
Type of CAD	
Chronic stable angina	53 (49.5)
Recent myocardial infarction	25 (23.4)
Unstable angina	17 (15.9)
Acute myocardial infarction	12 (11.2)
CCS functional classification of angina	
One	33 (30.8)
Two	49 (45.8)
Three	25 (23.4)

Values are shown as either mean ± standard deviation, or n (%)

*BMI* body mass index, *CAD* coronary artery disease, *CCS* Canadian Cardiovascular Society

Further cardiovascular description included image studies like echocardiography measurements and angiographic CAD characterization, results are summarized in Table 2. Mean EAT thickness was 4.6 mm and no significant functional abnormalities were observed. Coronary angiography showed damage in more than two coronary arteries in most of cases, being the anterior descendent coronary artery mainly affected and low degree of complexity was most common, as evaluated by Syntax score calculation.

**Table 2:** Echocardiographic measures and angiographic CAD characteristics

Echocardiographic measures	
EAT thickness (mm)	4.6 ± 1.99
LVEF (%)	53.5 ± 11.55
E/A	1.1 ± 0.76
E/Ea	11.0 ± 4.93
Ventricle mass (g/m^2^)	147.6 ± 47.89
Angiographic CAD characteristics	
Type of coronary artery affected	
More than two coronary arteries^a^	62 (58.0)
Left anterior descending artery + right coronary artery	20 (18.7)
Left anterior descending artery + circumflex artery	16 (14.9)
Left anterior descending artery alone	9 (8.4)
Syntax score complexity	
Low	69 (64.5)
Intermediate	16 (14.9)
High	22 (20.6)

*EAT* epicardial adipose tissue, *LVEF* left ventricle ejection fraction, *E/A* refers to the E/A ratio where *E* early diastole wave and *A* end-diastole (atrial contraction) wave, *E/Ea* refers to the index of left atrial pressure

^a^It consistently includes the left anterior descending artery. Values are shown as either mean ± standard deviation, or n (%). Syntax score of complexity of CAD, assessed as low (0–22), intermediate (23–32) and high (≥ 33)

**Fig. 1:**
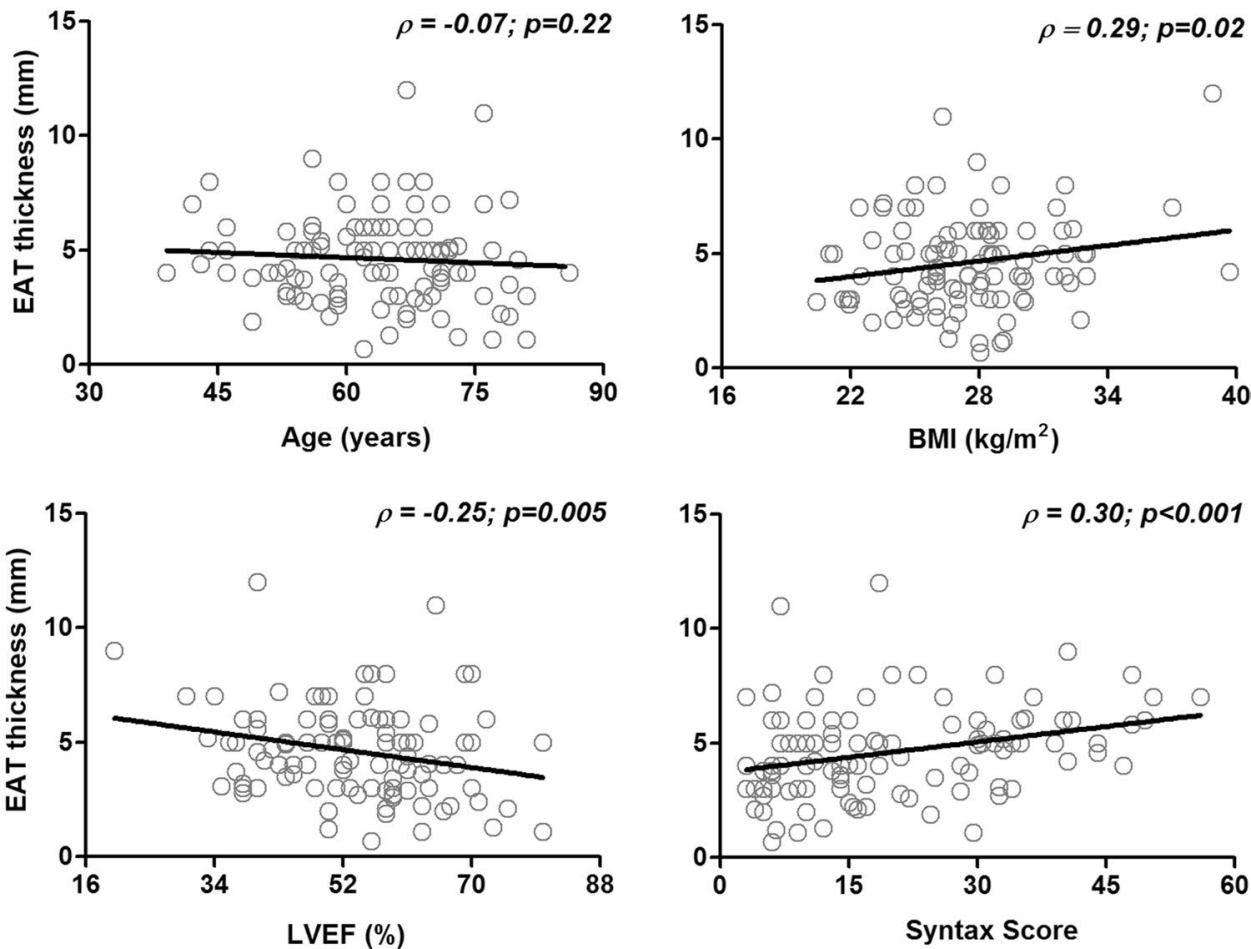
Relation of EAT thickness with cardiovascular risk markers. The graphics show distribution and Pearson's correlation analysis of EAT with age, BMI, LVEF y and Syntax score.* Abbreviations* *EAT* epicardial adipose tissue, *BMI* body mass index, *LVEF* left ventricle ejection fraction. *p* value, indicates statistical significance. Artwork was created in GraphPad Prism 5.0

The ability of EAT thickness to reflect cardiovascular risk was initially estimated based on its relation with some markers already studied in previous reports (Fig. 1). We observed that EAT significantly correlated with the complexity of coronary arteries and BMI, while negatively correlated with LVEF.

**Fig. 2:**
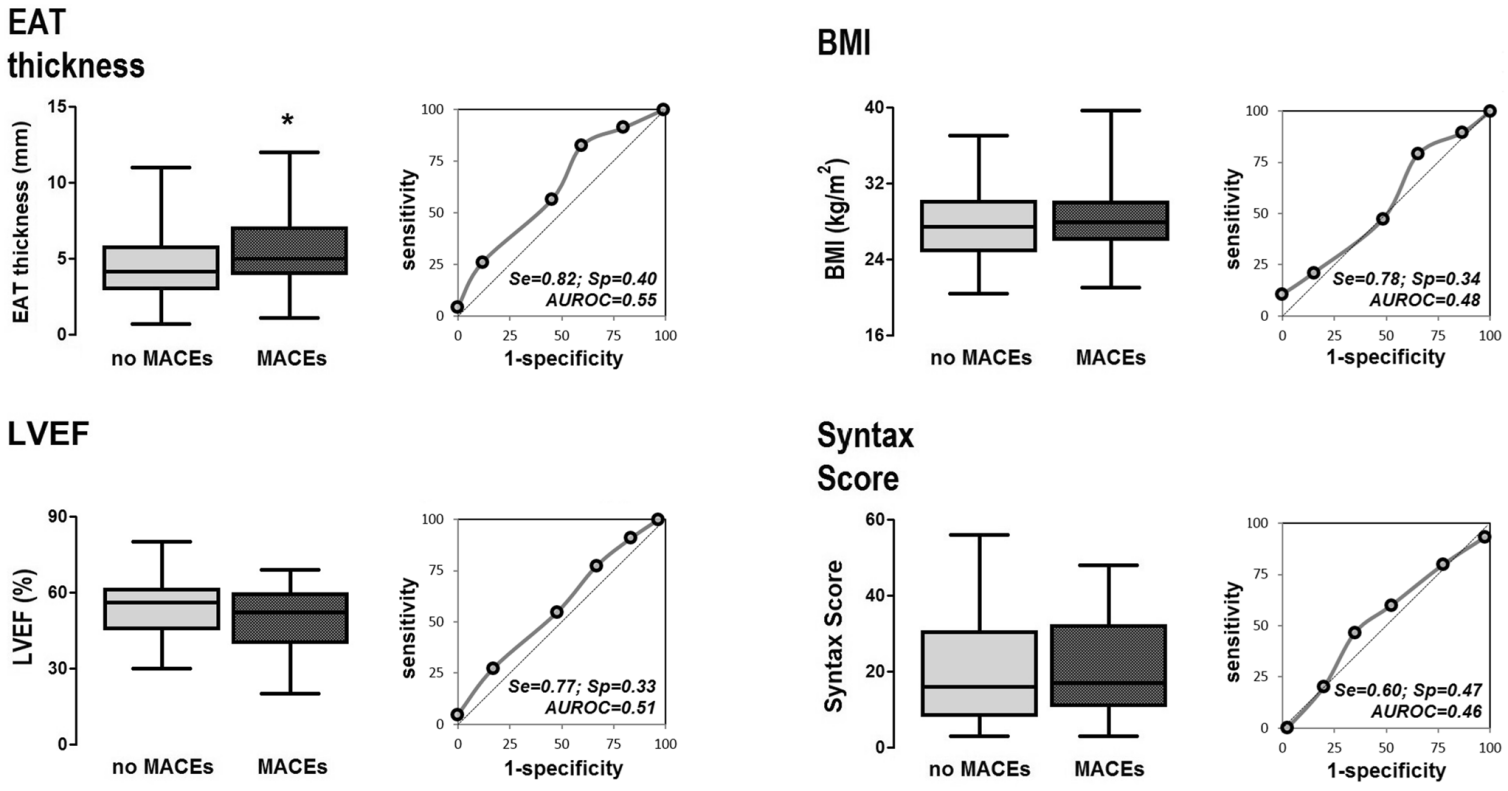
Comparative ability of EAT thickness to predict MACE’s. The boxplots show the values of EAT thickness, BMI, LVEF and Syntax score, as divided by the development of MACE’s. The ROC curve indicates the area under the curve (AUROC’s), and values (%) of sensitivity (Se) and specificity (Sp). *Statistically significant, p < 0.05. *Abbreviations* *EAT* epicardial adipose tissue, *BMI* body mass index, *LVEF* left ventricle ejection fraction. Artwork was created in GraphPad Prism 5.0

To evaluate whether EAT thickness might predict development of MACE’s, the study population was followed up for a mean time of 16 months after the coronary intervention (15.94 ± 3.6 months, ranging from 12 up to 24 months), where the number of MACE's were registered. During this time period, 23 MACE's were recorded: two cardiovascular related deaths, 14 hospitalizations due to unstable angina, five cases who required coronary catheterism, two intra-stent re-stenosis and five cases with concomitant heart failure requiring hospital attention. According to comparative analysis (Table 3) the group that developed MACE’s characterized by significantly higher EAT thickness and lower E/A measures; as well as higher prevalence of diabetes mellitus and chronic stable angina, most of them classified as type II CCS. Likewise, diagnostic performance of EAT to discriminate MACE's was comparable to LVEF, both AUROC’s > 0.5; and higher sensitivity than Syntax score; while low specificity values were obtained for all markers (Fig. 2).

**Table 3:** Comparative characteristics according to MACE’s

	Non-MACE’s (n = 84)	MACE’s (n = 23)
Age (years)	63.1 ± 9.17	65.2 ± 11.39
Male [n (%)]	69 (82.1)	17 (73.9)
BMI	27.4 ± 3.43	28.6 ± 4.75
Smoke	44 (52.4)	8 (34.8)
Co-morbidity		
High blood pressure	62 (73.8)	15 (65.2)
Dyslipidemia	49 (58.3)	16 (69.6)
Type 2 diabetes mellitus	37 (44.0)	17 (73.9)*
Obesity	18 (21.4)	7 (30.4)
History of CAD	38 (45.2)	14 (60.9)
Type of CAD		
Chronic stable angina	37 (44.0)	16 (69.6)*
Recent myocardial infarction	22 (26.2)	3 (13.0)
Unstable angina	15 (17.9)	2 (8.7)
Acute myocardial infarction	10 (11.9)	2 (8.7)
CCS classification of angina		
One	31 (36.9)	2 (8.7)*
Two	29 (34.5)	20 (87.0)*
Three	24 (28.6)	1 (4.3)*
Echocardiographic measures		
EAT thickness (mm)	4.4 ± 1.81	5.3 ± 2.43*
LVEF (%)	54.2 ± 11.35	50.3 ± 12.07
E/A	1.14 ± 0.817	0.77 ± 0.301*
E/Ea	10.8 ± 5.30	12.0 ± 3.22
Ventricle mass (g/m^2^)	150.5 ± 48.0	139.8 ± 48.1
Angiographic CAD characteristics		
Type of coronary artery affected		
More than two coronary arteries^a^	46 (54.8)	16 (69.6)
LADA artery + RCA	19 (22.6)	1 (4.3)
LADA + CA	11 (13.1)	5 (21.8)
LADA alone	8 (9.5)	1 (4.3)
Syntax score complexity		
Low	53 (63.1)	16 (69.6)
Intermediate	14 (16.7)	2 (8.6)
High	17 (20.2)	5 (21.8)

Values are shown as either mean ± SD, or n (%).Syntax score of complexity of CAD, assessed as low (0–22), intermediate (23–32) and high (≥ 33)

*BMI* body mass index, *CAD* coronary artery disease, *CCS* Canadian Cardiovascular Society, *EAT* epicardial adipose tissue, *LVEF* left ventricle ejection fraction, *E/A* refers to the E/A ratio where *E* early diastole wave and *A* end-diastole (atrial contraction) wave, *E/Ea* refers to the index of left atrial pressure, *LADA* left anterior descending artery, *RCA* right coronary artery, *CA* circumflex artery

*p < 0.05 two-way test for unequal variances

^a^Consistently includes the LADA

**Fig. 3:**
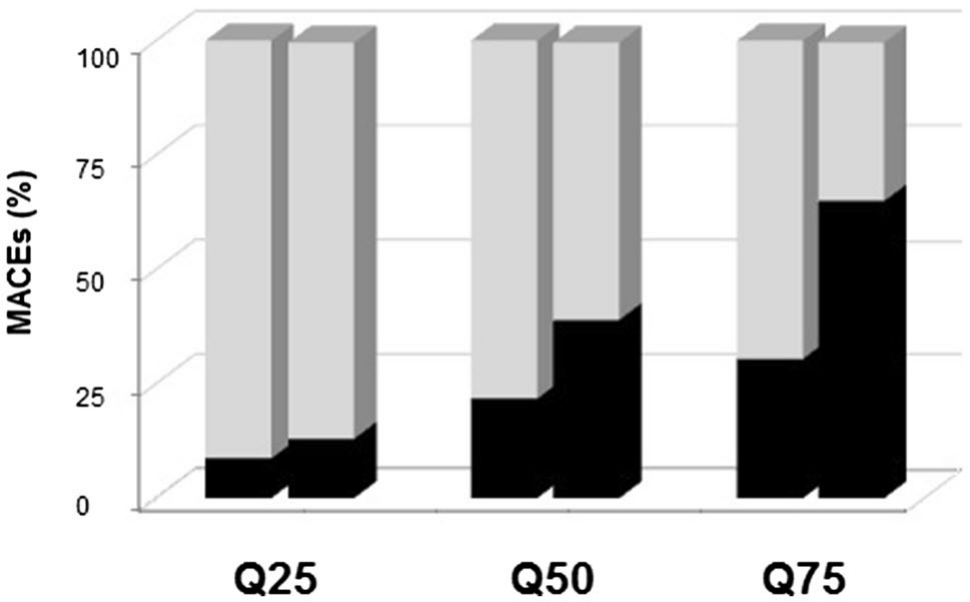
Range distribution of MACE’s according to location of EAT measure. Bars represent the percentage of MACEs identified at every quartile (Q), when EAT thickness was selectively measured over the right ventricle free wall (p25 = 3; p50 = 4.6, p75 = 6) or when measure of complete bulk of EAT was performed (p25 = 3.6; p50 = 11, p75 = 19). *Abbreviation* *MACE’s* major adverse cardiac events. Artwork was created in Excel

In this study, measurements of EAT were performed at several locations in order to test whether it may influence the ability to predict MACEs. Therefore, quartile comparison between values of EAT thickness measures of selective EAT lying over the right ventricle free wall, and measures as complete EAT, was performed (Fig. 3). We observed that measure of complete EAT provides a better discrimination range for MACE's than measure of the EAT lying over the right ventricle free wall.

Finally, adjusted risk for MACE's development attributable to EAT measures at both locations, as well as other potential risk factors, were further compared in a logistic regression analysis (Table 4). The models that included complete EAT measure resulted in a statistically significant increase of almost 4-times (RR = 3.91; 95% CI 1.01–15.08; p = 0.04) the risk of MACE's when complete bulk of EAT ≥ 4.6 cm.

**Table 4:** Logistic regression analysis of MACE’s

	OR	95% CI	*p*
Model 1a. EAT-complete*	3.41	1.01–10.56	**0.04**
Model 1b. EAT-complete, adjusted by type of CAD	3.74	1.08–12.88	**0.04**
Model 1c. EAT-complete, adjusted by T2DM and HBP	3.91	1.01–15.08	**0.04**
Model 1d. EAT-right ventricle^a^	1.90	0.72–5.02	0.19
Model 2. Syntax score	1.43	0.55–3.73	0.46
Model 3. BMI	1.13	0.42–3.00	0.80
Model 4. LVEF	1.09	0.41–2.84	0.85

Bold values indicate statistical significance

All models are shown after sex- and age- adjustment

Median cutoff values were: complete EAT measure 4.6 cm, EAT measured over the right ventricle 4.6 cm, Syntax score 16, BMI 28 and LVEF 55%

*MACE’s* major adverse cardiac events, *EAT* epicardial adipose tissue, *CAD* coronary artery disease, *T2DM* type 2 diabetes mellitus, *HBP* high blood pressure, *BMI* body mass index, *LVEF* left ventricle ejection fraction

*Measure of the complete EAT

^a^Measure of the EAT lying over the free wall of the right ventricle

## Discussion

Our study compared the ability of EAT thickness to predict MACE’s in patients with CAD. We found that EAT thickness significantly associated with MACE’s development; with a higher risk than the other markers tested.

EAT constitutes a type of VAT with intimate relation with the heart and coronary arteries, which owns unique characteristics regarding adipocyte's hypertrophy, biochemical and metabolic activities [22]. EAT is taught to exert pro-atherogenic effects acting as a source of pro-inflammatory mediators in a close proximity to coronary arteries and myocardium. Indeed, the relationship between EAT with coronary atherogenesis, plaque volume and stability has been described, as well as its predicting ability for clinical outcome and MACE’s [11, 23–25]. Consistently, in the present study higher values of EAT were observed in the group that developed MACE’s during the follow up period; whereas factors like lower E/A measures, diabetes mellitus and the type of CAD may also be involved. We observed that EAT maintained as independent risk factor for MACE’s after weighing potential interactions by logistic regression analysis.

In addition, EAT significantly related with cardiometabolic risk markers like BMI, LVEF and the degree of complexity of coronary arteries, reflected by the Syntax score; being consistent with previous reports also describing MACE’s predicting ability of such markers [11, 14, 25, 26]. However, the pathophysiological pathways leading cardiovascular risk underlying to each marker may be different. For example, BMI may be linked to low-grade inflammation and the “obesity paradox” risk phenomenon [17, 18]; while LVEF may be associated to myocardial injury, ventricular arrhytmia, pump failure with activation of renin-angiotensin-aldosterone and adrenergic systems leading to negative myocardial remodeling and deterioration in contractile function [15] and coronary severity may influence due to the degree of vascular damage, although the relation between coronary stenosis with plaque stability or future MACE’s is still debatable [16].

Therefore, we specifically compared EAT’s predicting ability of MACE's versus BMI, LVEF and complexity of coronary arteries in a study population with CAD, owning demographic characteristics and cardiovascular risk similar to previous reports [23]. In comparison with the other markers, only EAT thickness resulted significantly higher in patients who developed MACE’s; with an estimated risk for MACE's of almost four. Consistently, higher baseline values of EAT thickness have been predictive of MACE's, and independent association with similar risk values have been obtained in several studies [10, 11, 14, 23, 25]. Moreover, we found that EAT provided acceptable diagnostic performance for MACE’s, which was similar to LVEF and with higher sensitivity than Syntax score and BMI. The AUC in our study is lower than other reports [10]. To our knowledge, this is the first study comparing EAT thickness, BMI, LVEF and complexity of coronary arteries regarding their quantification, MACE’s association and diagnostic abilities.

In the present study, we performed echocardiographic measure of EAT thickness and used the median value to estimate the risk for developing MACE’s. However, there is a high variability in the way of measure EAT and the value considered of risk for MACE’s between studies. Some have used quartiles or 90th percentile of EAT basal measures, or even the EAT remodeling observed between basal measure and a 4-year subsequent measure; obtained through cardiac-CT with measures performed either as parallel, equidistant axial planes or full vertical length images of the heart [10, 25, 27]. Others have used the mean EAT, as echocardiographically determined, or receiver operating characteristics-obtained cutoff value best discriminating MACE’s; from measures of the thickest point at the echo-free space between the pericardium and the outer wall of the myocardium, performed usually at the free wall of the right ventricle from the parasternal long-axis views [11, 14]. Regardless the measurement method, EAT's predicting ability for MACE’s remains highly consistent and risk association values reported are very close to our result.

Moreover, we found that EAT showed a variable discrimination range for MACE's and different prediction ability, related to the location where EAT was measured. This finding has also been previously observed [27]. Although EAT measures on a single slice at specific level of the heart highly correlate with measures of total EAT burden [24], it seems that there is a location-dependent risk, at least observed in the prediction of coronary atherogenesis. According to studies and meta-analysis, EAT thickness measured at the left AV groove is associated with obstructive CAD risk [28, 29]; whereas scanty information exists regarding EAT's location-dependent risk for MACE’s. Currently, there is no guidelines or consensus regarding its clinical use of EAT measure. Some recommendations are based on its relation with MRI measures or with coronary angiography findings; moreover, echocardiographic cutoff values for increased EAT measures has been suggested [5]. However, echocardiographic method of EAT measure that best reflects its MACE’s prediction ability is to be defined.

The strength of our study are both, the performance analysis for MACE’s discrimination when echocardiographic EAT measurements are collected at different anatomical references; and the comparison of MACE’s prediction risk regarding other markers. However, major limitations of the present study includes: first, potential selection bias because patients were recruited from those referred for echocardiographic examination; second, no survival analysis at different time points for MACE’s was performed; and third, the relatively small number of patients and cardiac endpoints.

In conclusion, the ability of EAT thickness in predicting MACE’s was better than the other markers tested in a population with CAD. The influence of selective location of EAT measure over MACE’s discrimination performance deserves further study due to potential usefulness for cardiovascular outcome prediction.

## References

[1] Taguchi R, Takasu J, Itani Y, Yamamoto R, Yokoyama K, Watanabe S (2001). Pericardial fat accumulation in men as a risk factor for coronary artery disease. Atherosclerosis.

[2] Jeong JW, Jeong MH, Yun KH, Oh SK, Park EM, Kim YK (2007). Echocardiographic epicardial fat thickness and coronary artery disease. Circ J.

[3] Baker AR, Silva NF, Quinn DW, Harte AL, Pagano D, Bonser RS (2006). Human epicardial adipose tissue expresses a pathogenic profile of adipocytokines in patients with cardiovascular disease. Cardiovasc Diabetol.

[4] Peraza-Zaldívar JA, Suárez-Cuenca JA, Aceves-Millán R, Ixcamparij-Rosales C, Amezcua L, de Pérez-CabezaVaca R (2016). Pro-atherogenic mediators and subclinical atherogenesis are related to epicardial adipose tissue thickness in patients with cardiovascular risk. J Int Med Res.

[5] Bertaso AG, Bertol D, Duncan BB, Foppa M (2013). Epicardial fat: definition, measurements and systematic review of main outcomes. Arq Bras Cardiol.

[6] Salazar J, Luzardo E, Mejías JC, Rojas J, Ferreira A, Rivas-Ríos JR (2016). Epicardial fat: physiological, pathological, and therapeutic implications. Cardiol Res Pract.

[7] Yañez-Rivera TG, Baños-Gonzalez MA, Ble-Castillo JL, Torres-Hernandez ME, Torres-Lopez JE, Borrayo-Sanchez G (2014). Relationship between epicardial adipose tissue, coronary artery disease and adiponectin in a Mexican population. Cardiovasc Ultrasound.

[8] Tanındı A, Kocaman SA, Erkan AF, Uğurlu M, Alhan A, Töre HF (2013). Epicardial adipose tissue thickness is associated with myocardial infarction and impaired coronary perfusion. Anatol J Cardiol.

[9] Park JS, Choi SY, Zheng M, Yang HM, Lim HS, Choi BJ (2013). Epicardial adipose tissue thickness is a predictor for plaque vulnerability in patients with significant coronary artery disease. Atherosclerosis.

[10] Hajsadeghi F, Nabavi V, Bhandari A, Choi A, Vincent H, Flores F (2014). Increased epicardial adipose tissue is associated with coronary artery disease and major adverse cardiovascular events. Atherosclerosis.

[11] Chu CY, Lee WH, Hsu PC, Lee MK, Lee HH, Chiu CA (2016). Association of increased epicardial adipose tissue thickness with adverse cardiovascular outcomes in patients with atrial fibrillation. Medicine.

[12] Iacobellis G, Corradi D, Sharma AM (2005). Epicardial adipose tissue: anatomic, biomolecular and clinical relationships with the heart. Nat Clin Pract Cardiovasc Med.

[13] Iacobellis G, Ribaudo MC, Zappaterreno A, Iannucci CV, Leonetti F (2004). Relation between epicardial adipose tissue and left ventricular mass. Am J Cardiol.

[14] Park JS, Lee YH, Seo KW, Choi BJ, Choi SY, Yoon MH (2016). Echocardiographic epicardial fat thickness is a predictor for target vessel revascularization in patients with ST-elevation myocardial infarction. Lipids Health Dis.

[15] Im MS, Kim HL, Kim SH, Lim WH, Seo JB, Chung WY, Other Korea Acute Myocardial Infarction Registry (KAMIR) and Korea Working Group on Myocardial Infarction (KorMI) Investigators (2016). Different prognostic factors according to left ventricular systolic function in patients with acute myocardial infarction. Int J Cardiol.

[16] Pan HC, Sheu WH, Lee WJ, Lee WL, Liao YC, Wang KY (2015). Coronary severity score and C-reactive protein predict major adverse cardiovascular events in patients with stable coronary artery disease (from the Taichung CAD study). Clin Chim Acta.

[17] Kunimura A, Ishii H, Uetani T, Aoki T, Harada K, Hirayama K (2017). Impact of nutritional assessment and body mass index on cardiovascular outcomes in patients with stable coronary artery disease. Int J Cardiol.

[18] Thornqvist C, Gislason GH, Køber L, Jensen PF, Torp-Pedersen C, Andersson C (2014). Body mass index and risk of perioperative cardiovascular adverse events and mortality in 34,744 Danish patients undergoing hip or knee replacement. Acta Orthop.

[19] Montalescot G, Sechtem U, Achenbach S, Andreotti F, Arden C (2013). 2013 ESC guidelines on the management of stable coronary artery disease: the Task Force on the management of stable coronary artery disease of the European Society of Cardiology. Eur Heart J.

[20] Sianos G, Morel MA, Kappetein AP, Morice MC, Colombo A, Dawkins K (2005). The STX score: an angiographic tool grading the complexity of coronary artery disease. EuroIntervention.

[21] Hemingway H, Fitzpatrick NK, Gnani S (2004). Prospective validity of measuring angina severity with Canadian Cardiovascular Society class: the ACRE study. Can J Cardiol.

[22] Sacks HS, Fain JN (2007). Human epicardial adipose tissue: a review. Am Heart J.

[23] Gitsioudis G, Schmahl C, Missiou A, Voss A, Schüssler A, Abdel-Aty H (2016). Epicardial adipose tissue is associated with plaque burden and composition and provides incremental value for the prediction of cardiac outcome. A clinical cardiac computed tomography angiography study. PLoS ONE.

[24] Tran T, Small G, Cocker M, Yam Y, Chow BJ (2014). A single slice measure of epicardial adipose tissue can serve as an indirect measure of total epicardial adipose tissue burden and is associated with obstructive coronary artery disease. Eur Heart J Cardiovasc Imaging.

[25] Nakanishi K, Fukuda S, Tanaka A, Otsuka K, Jissho S, Taguchi H (2014). Persistent epicardial adipose tissue accumulation is associated with coronary plaque vulnerability and future acute coronary syndrome in non-obese subjects with coronary artery disease. Atherosclerosis.

[26] Chen J, Tang B, Lin Y, Ru Y, Wu M, Wang X (2016). Validation of the ability of Syntax and clinical Syntax scores to predict adverse cardiovascular events after stent implantation: a systematic review and meta-analysis. Angiology.

[27] Shmilovich H, Dey D, Cheng VY, Rajani R, Nakazato R, Otaki Y (2011). Threshold for the upper normal limit of indexed epicardial fat volume: derivation in a healthy population and validation in an outcome-based study. Am J Cardiol.

[28] Wu FZ, Chou KJ, Huang YL, Wu MT (2014). The relation of location-specific epicardial adipose tissue thickness and obstructive coronary artery disease: systemic review and meta-analysis of observational studies. BMC Cardiovasc Disord.

[29] Wang TD, Lee WJ, Shih FY, Huang CH, Chen WJ, Lee YT (2010). Association of epicardial adipose tissue with coronary atherosclerosis is region-specific and independent of conventional risk factors and intra-abdominal adiposity. Atherosclerosis.

